# Incidence, Characteristics, and Outcomes of Stroke in Pediatric Patients with Celiac Disease

**DOI:** 10.3390/life13071445

**Published:** 2023-06-26

**Authors:** Sima Vazquez, Akash Thaker, Bridget Nolan, Eris Spirollari, Kevin Clare, Steven Wolf, Patricia McGoldrick, Rolla Nuoman, Philip Overby, Fawaz Al-Mufti

**Affiliations:** 1 School of Medicine, New York Medical College, Valhalla, NY 10595, USA; athaker@student.nymc.edu (A.T.); espiroll@student.nymc.edu (E.S.); fawaz.al-mufti@wmchealth.org (F.A.-M.); 2 Department of Neurosurgery, Westchester Medical Center, Valhalla, NY 10595, USA; bnolan2@student.nymc.edu (B.N.); kclare@student.nymc.edu (K.C.); 3 Department of Neurology, Boston Children’s Health Physicians, New York, NY 10595, USA; steven.wolf@wmchealth.org (S.W.); patricia_mcgoldrick@bphphysicians.org (P.M.); rolla.nuoman@wmchealth.org (R.N.); 4 Department of Pediatric Neurology, Maria Fareri Children’s Hospital, Valhalla, NY 10595, USA; philip.overby@wmchealth.org; 5 Department of Neurology, Westchester Medical Center, Valhalla, NY 10595, USA

**Keywords:** pediatrics, acute ischemic stroke, celiac disease

## Abstract

(1) Background: Celiac disease (CD) can cause long-term inflammation and endothelial dysfunction and has been cited as a risk factor for acute ischemic stroke (AIS) in pediatric patients. However, the rate and outcomes of AIS in pediatric patients with CD has not been explored in a large population. Our objective is to explore the rate, severity, and outcomes of CD amongst pediatric AIS patients on a nationwide level. (2) Methods: The National Inpatient Sample (NIS) database was queried from 2016 to 2020 for pediatric patients with a principal diagnosis of AIS. Patients with a concurrent diagnosis of CD (AIS-CD) were compared to those without (AIS). Baseline demographics and comorbidities, clinical variables of severity, hospital complications, and the rates of tissue plasminogen activator (tPA) and mechanical thrombectomy were compared between the two groups. The main outcomes studied were mortality, discharge disposition, length of stay (LOS), and total hospital charges. (3) Results: Of 12,755 pediatric patients with a principal diagnosis of AIS, 75 (0.6%) had concurrent CD. There were no differences in the severity, discharge disposition, or mortality between the AIS-CD and AIS patients. Patients with AIS-CD were more likely to receive tPA at an outside hospital within 24 h of admission (*p* < 0.01) and more likely to undergo mechanical thrombectomy (*p* < 0.01) compared to the AIS patients. (4) Conclusions: CD patients made up only 0.6% of all pediatric AIS patients. No differences in the severity, mortality, or discharge disposition suggests a minimal to absent role of CD in the etiology of stroke. The CD-AIS patients were more likely to receive a tPA or undergo a mechanical thrombectomy; studies are needed to confirm the safety and efficacy of these interventions in pediatric patients.

## 1. Introduction

Celiac disease (CD) is a life-long autoimmune condition. It is associated with major histocompatibility complex class II genes and the alleles encoding the human leukocyte antigen molecules (HLA)-DQ2 and HLA-DQ8 [1]. Notably, these genetic and molecular configurations have far-reaching implications in the pathology of CD. Clinically, CD is typically recognized for its intestinal impacts. These enteropathic symptoms are predominantly triggered by the consumption of gluten, a common dietary protein, which subsequently enables gliadin protein-mediated autoimmune infiltration of the intestinal epithelial layer [1].

While this understanding of CD offers an essential overview, there is evidence supporting the role of celiac antibodies extending beyond the intestinal tract to other systemic pathologies [2,3,4,5,6,7]. Among these non-intestinal manifestations, the most well-known complication of CD is the dermatologic pathology of dermatitis herpetiformis. More strikingly, there also exists an extensive number of associations between CD and neurological disorders. For instance, neuropathy, dementia, brain atrophy, cerebellar ataxia, epilepsy with occipital calcifications, and several additional neurological diseases have been linked to CD [2,3,4,5,6,7]. Particularly, a unique subtype of cerebellar ataxia called gluten ataxia can be a manifestation of CD. This pathology is a sporadic cerebellar ataxia, marked by the presence of anti-gliadin antibody IgA, with no other discernable causes for the ataxia. Recent investigations have discovered that patients with gluten ataxia exhibit celiac-specific trans-glutaminase targeted IgA deposits in the intestinal mucosa as well as in the cerebral vasculature. These deposits in the cerebral vasculature are linked with a substantial loss of Purkinje cells, suggesting a possibility of CD-associated cerebrovascular pathology [8]. Further enhancing our understanding of these vascular CD manifestations, there have been several studies demonstrating an association between CD and endothelial dysfunction, accelerated atherosclerosis, atrial fibrillation, and a moderately increased risk of cardiovascular disease [2,3,4,5,6,7,9]. These correlations further uncover the systemic impacts of CD and allude to the presence of a broad array of pathophysiological links and disease mechanisms that are yet to be fully understood.

Among all the cerebrovascular events, it is noteworthy that acute ischemic stroke (AIS) is an uncommon insult in pediatric patients. In one prospective nationwide population-based study, the incidence of stroke in children was established to be 1.72 per 100,000 per year [10]. Among older children who presented with AIS, arteriopathy, cardiac disorders, and prothrombotic disorders were frequently found [10]. In addition to these associations, a link between CD and AIS has also been hypothesized [6]. There is evidence for the presence of IgA antibodies reacting with structures within the cerebral vasculature in patients with active CD [8]. These same antibodies have been confirmed to cross-react with transglutaminase, the principle active autoantigen implicated in CD [5]. This interplay of chronic inflammation, cross-reacting antibodies, and endothelial dysfunction may influence the increased rates of ischemic stroke, myocardial infarction, and thromboembolism observed in patients with CD [11]. 

Despite the existence of evidence for a link between CD and vascular disease, there has been little exploration of the associations and outcomes of pediatric patients with CD and AIS. One nationwide cohort study conducted in Sweden demonstrated that the excess risk of stroke in patients with CD was 24/100,000. Conversely, other research indicates little to no risk of AIS in pediatric patients with CD [12,13]. Therefore, this study aims to examine the incidence and outcomes of pediatric AIS patients with CD in a large nationwide sample. Our goal is to shed light on these associations and outcomes; thus, filling an important knowledge gap in our understanding of the interplay between CD and AIS.

## 2. Materials and Methods

### 2.1. Data Source and Patient Selection

The National Inpatient Sample (NIS) database is provided by the Healthcare Cost and Utilization Project (HCUP) and is the largest publicly available database with data from over seven million admissions per year from around the nation. The data variables include demographic characteristics, hospital and regional information, inpatient diagnoses and procedures, medications, and discharge disposition. The NIS is publicly available and contains no identifiable information. Therefore, approval by an institutional review board was not required for this study. The NIS 2016–2020 was queried for patients under the age of 18 with a principal diagnosis of AIS using the International Classification of Disease 10th Edition (ICD10) code I63. A cohort of patients with a concurrent diagnosis of CD (ICD10 K90.0) was created. Patients presenting with AIS with celiac (AIS-CD) were compared to those without celiac (AIS).

### 2.2. Data Characteristics and Outcomes Measured

The demographic characteristics, such as age, sex, insurance status, race, and socioeconomic status were described. As defined by the HCUP, the Q1 median income category reflects the lowest 25th percentile median income category in the United States. Comorbidities, such as hypertension, long-term anticoagulation or antiplatelet therapy, nutritional anemia, and thrombophilia, were compared between the AIS-CD and AIS patients. Acute stroke indices, such as aphasia, hemiplegia, mechanical ventilation, cerebral edema, coma, and herniation, were compared and summed to create a stroke severity scale. Inpatient complications and treatment with a tissue plasminogen activator (tPA) or mechanical thrombectomy were analyzed. The outcome measures studied were mortality and discharge disposition. Other outcomes studied included variables of healthcare resource utilization, such as the length of stay (LOS) and the total hospital charges. Prolonged LOS was defined as a LOS greater than the 75th percentile of the entire cohort, or greater than 28 days. An increased hospital charge was defined as hospital charges greater than the 75th percentile of the entire cohort, or greater than USD 441,670. 

### 2.3. Statistical Analysis

Categorical variables were compared using Pearson’s chi-squared test. Continuous variables were evaluated for normality using the Kolmogorov–Smirnov Test. Normally and non-normally distributed continuous variables were tested using the Student’s *t* test and Mann–Whitney U test, respectively. Presentation, interventions, complications, and outcomes were compared between AIS and AIS-CD patients. A univariate and multivariate logistic regression was performed to identify the independent predictors of mortality, prolonged LOS, and increased total charges. Statistical Product and Service Solutions (SPSS) Statistical Software was used for the analysis, and the statistical significance was set at <0.05 (IBM Corp. Released 2020. IBM SPSS Statistics for Windows, Version 28.0. Armonk, NY, USA: IBM Corp).

### 2.4. Data Availability Statement

All data utilized in this study are available upon reasonable request submitted to the corresponding author. A completion of onboarding procedures specified by the Healthcare Cost and Utilization Project will be required.

## 3. Results

### 3.1. Demographics and Comorbidities

We conducted an extensive evaluation of the pediatric patients who were admitted due to AIS during the period spanning from 2016 to 2020. Within this time frame, there were 12,755 pediatric patients admitted for AIS. Among this cohort of pediatric AIS patients, 75 patients (0.6%) had a concurrent diagnosis of CD. The age distribution among the AIS-CD patients was 8.00 years, with a standard deviation of 6.82 years. Comparatively, the average age of the patients with AIS but without CD was slightly lower at 5.83 years, with a standard deviation of 6.34 years. In our comparative analysis of the AIS pediatric patients with vs. without CD, the patients with AIS-CD were more likely to be greater than 12 years old (OR 2.13 95% CI 1.34–3.38 *p* < 0.01), indicating an association between older pediatric age and concurrent AIS-CD diagnosis. Moreover, our analysis demonstrated that the AIS-CD patients were more likely to be Caucasian (OR 2.50 95% CI 1.55–4.05 *p* < 0.01), possibly implying a significant racial predisposition in the link between AIS and CD. The analysis further revealed a notable association between long-term anticoagulation or antiplatelet therapy and AIS-CD patients (OR 3.76 95% CI 2.13–6.66 *p* < 0.01), compared to non-CD patients. However, in examining the other demographic parameters, such as sex, income, or insurance status, our results did not indicate any significant differences between the AIS-CD group and the group suffering from AIS alone. Furthermore, upon investigation of other comorbid conditions, including hypertension, nutritional anemia, or thrombophilia, no significant differences were found between the two groups (Table 1).

### 3.2. Hospital Course

We also explored the various acute stroke indices in both the AIS and AIS-CD patient populations. There were no significant differences observed in terms of the critical symptoms typically associated with acute stroke, such as aphasia, which refers to any difficulties with language, and hemiplegia, defined as paralysis on one side of the body. Additionally, the need for mechanical ventilation, often required in more severe stroke cases, also did not show any notable difference between the AIS and AIS-CD groups. We compared the stroke severity scale, defined by the intensity and scale of the neurological deficits, and no significant differences were detected. These findings may suggest that the presence of CD does not appear to have a clear influence on the acute presentation or severity of stroke in pediatric patients (Table 2). 

We analyzed and compared the acute management interventions and systemic complications for the AIS and AIS-CD patient groups. Patients with AIS-CD were more likely to receive a tPA, an enzyme involved in the breakdown of blood clots, at an outside hospital within the first 24 h following admission (OR 7.80 95% CI 3.09–19.70 *p* < 0.01). Another intervention that showed a significant increase in incidence among the AIS-CD patients compared to AIS alone was mechanical thrombectomy, a procedure used to remove a blood clot from a vessel in the brain (OR 4.14 95% CI 1.66–10.36 *p* < 0.01). In terms of gastrointestinal interventions, percutaneous endoscopic gastrostomy was more likely in AIS-CD compared to AIS patients (OR 3.04 95% CI 1.72–5.36 *p* < 0.01). These findings suggest a higher propensity for interventions in the management of AIS for patients with CD. Regarding complications, our analysis demonstrated a significant association with acute kidney injury (OR 3.44 95% CI 2.12–5.57 *p* < 0.01) and sepsis (OR 7.32 95% CI 4.36–12.29 *p* < 0.01) in the AIS-CD cohort. There were no notable differences in terms of the occurrence of urinary tract infections, pneumonia, or deep venous thrombosis between the two groups, suggesting that while pediatric AIS patients with CD were not necessarily predisposed to an increase in these specific systemic complications, they may still have had a greater likelihood of sepsis and acute kidney injury (Table 3).

### 3.3. Outcomes

In evaluating the outcomes of the AIS and AIS-CD patients, we found the average duration of hospital stay for the AIS-CD patients to be significantly longer, at 46.93 days, with a standard deviation of 46.83 days. In comparison, the mean LOS for pediatric AIS patients, without a concurrent diagnosis of CD, was significantly shorter, at 25.04 days, with a standard deviation of 38.89 days. Moreover, the patients with AIS-CD demonstrated a greater likelihood for a prolonged LOS (OR 3.61 95% CI 2.29–5.69 *p* < 0.01), indicating a possible significant influence of concurrent CD on the hospitalization duration of AIS pediatric patients. In terms of financial implications, we also analyzed the average total hospital charges for both groups. The average total charges for the AIS-CD group were significantly higher, at USD 737,694.98, with a standard deviation of USD 630,184.09. Comparatively, for the AIS patients, the mean hospital charges were lower, at USD 478,549.04, with a standard deviation of USD 927,106.93. AIS-CD was associated with increased total hospital charges (OR 3.52 95% CI 2.24–5.56 *p* < 0.01), possibly signifying a considerable economic burden associated with the co-occurrence of AIS and CD. There were no significant differences between the two groups in terms of the discharge disposition or mortality rates (Table 4). 

On the multivariate regression analysis, CD was independently associated with a prolonged LOS (OR 5.17 95% CI 3.16–8.45 *p* < 0.01). Additionally, a stroke severity scale score greater than one was also independently associated with an extended LOS (OR 2.54 95% CI 2.32–2.78 *p* < 0.01). Increasing age (OR 0.94 95% CI 0.93–0.94 *p* < 0.01) and stroke treatment with a tPA or a mechanical thrombectomy (OR 0.31 95% CI 0.20–0.47 *p* < 0.01) were negatively associated with a prolonged LOS. A stroke severity scale score greater than one was the only factor associated with mortality (OR 3.30 95% CI 2.89–3.78 *p* < 0.01). Older AIS patients (OR 0.96 95% CI 0.95–0.96 *p* < 0.01) and patients who received stroke treatment with a tPA or a mechanical thrombectomy (OR 0.14 95% CI 0.06–0.33 *p* < 0.01) were at lower odds of inpatient death. A stroke severity scale score greater than one (OR 3.16 95% CI 2.88–3.46 *p* < 0.01) and CD (OR 4.76 95% CI 2.93–7.73 *p* < 0.01) were associated with increased total hospital charges, while increasing age (OR 0.96 95% CI 0.95–9.96 *p* < 0.01) and a tPA or a mechanical thrombectomy (OR 0.35 95% CI 0.24–0.51 *p* < 0.01) were associated with decreased total charges (Table 5).

## 4. Discussion

In our study, we explore the incidence, comorbidities, and outcomes of pediatric patients with CD who present with an AIS throughout the nation. CD has been cited as a risk factor for ischemic stroke and venous thromboembolism throughout the literature [9,14,15,16]. The proposed pathophysiologic mechanisms center around the presence of tissue transglutaminase antibodies and antibody-mediated inflammation [6,8,13,14,15]. Studies have also correlated low vitamin B12, low folic acid levels, and high homocysteine levels, with hypercoagulability [14,15,17]. Our nationwide analysis found that patients with CD made up only 0.6% of the pediatric patients presenting with AIS. This compares to a CD prevalence of 0.7% in the general population [18]. Furthermore, we show no differences in the severity, mortality, or discharge disposition between those with celiac and those without. 

Studies with large populations exploring the association between CD and pediatric stroke show a low incidence of AIS in CD children [12,13]. One study found that 2 out of 76 (2.26%) pediatric AIS patients and 2 out of 102 (1.96%) healthy children had positive serum tissue transglutaminase antibodies; this difference was not statistically significant [12]. A multi-institutional study of over 28,000 patients with biopsy-confirmed CD found an absolute risk of stroke of 267/100,000 person-years [13]. This study did find an increased risk for stroke in patients with CD compared to sex- and age-matched controls, with the highest risk in the first year following diagnosis [13]. Other studies also show an increased risk in CD patients who are under-treated or unable to uphold a gluten-free diet; whereby, tissue transglutaminase antibodies and chronic inflammation can lead to hypercoagulability or endothelial dysfunction [13,19,20]. One study found no increased risk of AIS in CD patients after more than 5 years of follow-up after diagnosis [13]. When treatment compliance is considered, well-treated CD patients show a negligible risk of AIS and venous thromboembolism [13,19].

Despite similar severity profiles upon presentation, the AIS-CD patients had much higher odds of receiving tPA or undergoing mechanical thrombectomy than AIS patients without CD. In a social setting that promotes familial involvement and monitoring of their child’s symptoms, this could be due to heightened parental awareness of any acute manifestation in a child with chronic disease, leading to an earlier presentation [21,22]. This increase in treatment may also be due to a lower physician threshold for administering a tPA or undergoing a mechanical thrombectomy in pediatric patients with a risk factor for an AIS. Since this population was excluded from the clinical trials, there remains little guidance on the optimal treatment of AIS in children [23]. It is also possible that the pediatric AIS patients without CD had other comorbid conditions that rendered them as ineligible for acute AIS treatment. In a large national prospective study conducted in Canada, acute illnesses were present in 48% of the children with an AIS [10]. These included sepsis, meningitis, or major trauma [10]. This study also found that 28% of older children with an AIS had other congenital or acquired cardiac disorders [10]. Acute illness and underlying cardiac comorbidity may increase the hesitancy in administering thrombolytics or pursuing endovascular therapy due to the risks of bleeding, infection, and limited studies in these patient populations. 

Without clear guidelines, AIS characteristics and patient selection are weighed heavily, and physicians may have a high threshold to pursue endovascular therapy in the setting of pediatric stroke without a clear etiology [18]. Similar outcomes between the AIS-CD and AIS patients suggest the safety and efficacy of a tPA and mechanical thrombectomy and suggest a minimal to absent role of CD in the etiology of stroke. Randomized controlled trials are necessary to assess the safety and efficacy of a tPA and mechanical thrombectomy in this population.

Our study found an 11% inpatient mortality rate in all pediatric AIS patients. This is consistent with the rates of inpatient mortality cited in other studies with large populations [10,24]. Of note, an underlying diagnosis of CD was not associated with mortality. This may be mediated by the increased rates of tPA and mechanical thrombectomy use in this population, as an early intervention for stroke is known to improve outcomes [10]. 

Pediatric AIS patients with CD had increased healthcare resource utilization, measured via the LOS and total healthcare charges. Healthcare resource utilization has become an important metric to discuss the value of care, effective patient care, and efficient resource allocation. Data from the Global Burden of Disease 2017 study showed that stroke was the most burdensome neurological disorder in the United States [25]. Although patients with CD may require more intensive treatment and multi-system management, longer hospital stays are known to increase the risk of urinary tract infection, pneumonia, sepsis, and deep venous thrombosis. Furthermore, patients with AIS and CD may be in the early stage of disease or in under-treated disease, making them vulnerable to malnutrition and further increasing the risk for nosocomial infection [26]. Our results provide data that generate hypotheses for future studies to explore efficient healthcare resource utilization while maximizing patient care for children with AIS and an underlying comorbid systemic disease.

As a database study, this study has inherent limitations. It is retrospective and correlative in nature; causation cannot be determined. However, we hope these results serve to show the incidence and outcomes of patients with AIS and CD throughout the nation. Although the NIS performs audits on participating institutions, there may be heterogeneity in the use of the ICD10 codes throughout the nation. There may, therefore, be under- or over-reporting of diagnoses and procedures. Important to the topic of study, the population was selected using ICD codes. It is not specified whether the diagnosis of CD required biopsy confirmation or not. Additionally, the time of diagnosis, time on a gluten-free diet, level of diet compliance, and the serologic markers or inflammatory laboratory values are unknown. These factors may play a role in the risk and severity of AIS, as under-treated CD patients may have chronic inflammation and endothelial dysfunction and should be further explored in more detail. Further studies are warranted to analyze the association between active tissue transglutaminase antibodies, the degree of intestinal mucosa atrophy, and the risk of AIS in pediatric patients. The NIS is an inpatient database; therefore, long-term outcomes, such as the rate of stroke recurrence, functional disability, and complications following discharge, are not available. 

## 5. Conclusions

CD patients made up only 0.6% of all pediatric AIS patients compared to a prevalence of 0.7% in the general population. No differences in the severity, mortality, or discharge disposition, suggests a minimal to absent role of CD in the etiology of stroke. The CD-AIS patients were eight times more likely to receive a tPA and four times more likely to undergo a mechanical thrombectomy. Additional studies are needed to confirm the safety and efficacy of these interventions in pediatric patients. 

## Figures and Tables

**Table 1:** Demographics and Comorbidities of Pediatric AIS Patients with and without CD.

Variables	Total AIS (12,755)	OR (95% CI)	*p* Value
Age > 12	3055 (24%)	2.13 (1.34–3.38)	<0.01
Female	5505 (43.2%)	0.88 (0.55–1.39)	0.64
Caucasian	5685 (44.6%)	2.50 (1.55–4.05)	<0.01
Q1 median income	3830 (30%)	0.85 (0.51–1.41)	0.53
Medicaid insurance	6625 (51.9%)	0.62 (0.39–0.98)	0.05
Private insurance	5125 (40.2%)	0.99 (0.62–1.58)	1.00
Hypertension	1140 (8.9%)	1.57 (0.81–3.07)	0.22
Anticoagulation/Antiplatelet	805 (6.3%)	3.76 (2.13–6.66)	<0.01
Nutritional anemia	425 (3.3%)	2.09 (0.84–5.19)	0.11
Thrombophilia	420 (3.3%)	2.11 (0.85–5.26)	0.10

Chi-squared analysis comparing the baseline demographics, socioeconomic status, and comorbidities. The odds ratio (OR) is in reference to the AIS-CD group.

**Table 2:** Stroke Severity Clinical Variables.

Variables	Total AIS (12,755)	OR (95% CI)	*p* Value
Aphasia	1030 (8.1%)	0.812 (0.327–2.017)	0.69
Hemiplegia	3290 (25.8%)	1.442 (0.891–2.334)	0.15
Mechanical ventilation	3315 (26%)	1.427 (0.882–2.31)	0.15
Stroke severity scale > 1	7400 (58%)	1.086 (0.683–1.726)	0.82

Chi-squared analysis comparing the stroke severity clinical variables. The odds ratio (OR) is in reference to the AIS-CD group.

**Table 3:** Hospital Course of Pediatric AIS Patients with and without CD.

Variables	Total AIS (12,755)	OR (95% CI)	*p* Value
tPA at an outside hospital within 24 h of admission	120 (0.9%)	7.80 (3.09–19.70)	<0.01
Mechanical thrombectomy	220 (1.7%)	4.14 (1.66–10.36)	<0.01
Urinary tract infection	475 (3.7%)	1.86 (0.75–4.62)	0.21
Acute kidney injury	1635 (12.8%)	3.44 (2.12–5.57)	<0.01
Pneumonia	950 (7.4%)	0.89 (0.36–2.20)	0.84
Deep venous thrombosis	580 (4.5%)	1.50 (0.61–3.74)	0.40
Sepsis	620 (4.0%)	7.32 (4.36–12.29)	<0.01
Percutaneous endoscopic gastrotomy	980 (7.7%)	3.04 (1.72–5.36)	<0.01

Chi-squared analysis showing the hospital course and complications of AIS patients. The odds ratio (OR) is in reference to the AIS-CD group.

**Table 4:** Outcomes of Pediatric AIS Patients with and without CD.

Variables	Total AIS (12,755)	OR (95% CI)	*p* Value
Prolonged LOS	3090 (24.2%)	3.61 (2.29–5.69)	<0.01
Discharge to home	7780 (61%)	0.96 (0.60–1.52)	0.91
Transfer to short-term hospital	960 (7.5%)	0.88 (0.35–2.18)	0.83
Inpatient death	1405 (11%)	0.58 (0.23–1.43)	0.27
Increased total charges	3145 (24.7%)	3.52 (2.24–5.56)	<0.01

Chi-squared analysis of the outcomes in AIS patients. The odds ratio (OR) is in reference to the AIS-CD group. Prolonged LOS and increased total charges are defined as a LOS and charges greater than the 75th percentile of the entire cohort. The odds ratio (OR) is in reference to the AIS-CD group.

**Table 5:** Predictors of Outcomes in Pediatric AIS Patients.

Variables	Prolonged LOS		Mortality		Increased Total Charges	
	OR (95% CI)	*p* Value	OR (95% CI)	*p* Value	OR (95% CI)	*p* Value
Age	0.94 (0.93–0.94)	<0.01	0.96 (0.95–0.96)	<0.01	0.96 (0.95–0.96)	<0.01
Female	0.99 (0.91–1.08)	0.83	1.09 (0.97–1.22)	0.15	1.05 (0.96–1.14)	0.27
Stroke severity scale > 1	2.54 (2.32–2.78)	<0.01	3.30 (2.89–3.78)	<0.01	3.16 (2.88–3.46)	<0.01
CD	5.17 (3.16–8.45)	<0.01	0.66 (0.26–1.66)	0.38	4.76 (2.93–7.73)	<0.01
tPA or mechanical thrombectomy	0.31 (0.20–0.47)	<0.01	0.14 (0.06–0.33)	<0.01	0.35 (0.24–0.51)	<0.01

Multivariate logistic regression for outcomes. Prolonged LOS and increased total charges are defined as a LOS and charges greater than the 75th percentile of the entire cohort. The odds ratio (OR) is in reference to the AIS-CD group. CD = celiac disease.

## Data Availability

The NIS is a publicly available database. All data utilized are available upon reasonable request from the corresponding author and appropriate onboarding measures as defined by the HCUP.
